# Epigenetic Mechanisms in Irritable Bowel Syndrome

**DOI:** 10.3389/fpsyt.2020.00805

**Published:** 2020-08-14

**Authors:** Swapna Mahurkar-Joshi, Lin Chang

**Affiliations:** G. Oppenheimer Center for Neurobiology of Stress and Resilience, Division of Digestive Diseases, Department of Medicine at UCLA, Los Angeles, CA, United States

**Keywords:** irritable bowel syndrome, IBS, epigenetics, visceral hypersensitivity, DNA methylation, microRNA, histone modifications, long non-coding RNA

## Abstract

Irritable bowel syndrome (IBS) is a brain-gut axis disorder characterized by abdominal pain and altered bowel habits. IBS is a multifactorial, stress-sensitive disorder with evidence for familial clustering attributed to genetic or shared environmental factors. However, there are weak genetic associations reported with IBS and a lack of evidence to suggest that major genetic factor(s) contribute to IBS pathophysiology. Studies on animal models of stress, including early life stress, suggest a role for environmental factors, specifically, stress associated with dysregulation of corticotropin releasing factor and hypothalamus-pituitary-adrenal (HPA) axis pathways in the pathophysiology of IBS. Recent evidence suggests that epigenetic mechanisms, which constitute molecular changes not driven by a change in gene sequence, can mediate environmental effects on central and peripheral function. Epigenetic alterations including DNA methylation changes, histone modifications, and differential expression of non-coding RNAs (microRNA [miRNA] and long non-coding RNA) have been associated with several diseases. The objective of this review is to elucidate the molecular factors in the pathophysiology of IBS with an emphasis on epigenetic mechanisms. Emerging evidence for epigenetic changes in IBS includes changes in DNA methylation in animal models of IBS and patients with IBS, and various miRNAs that have been associated with IBS and endophenotypes, such as increased visceral sensitivity and intestinal permeability. DNA methylation, in particular, is an emerging field in the realm of complex diseases and a promising mechanism which can provide important insights into IBS pathogenesis and identify potential targets for treatment.

## Introduction

Irritable bowel syndrome (IBS) is a complex condition characterized by alterations of bidirectional brain-gut interactions affecting gastrointestinal (GI) function. It is a widely prevalent disorder affecting about 5% to 11% of general population and occurs in children and adults and in men and women although it is considered a female-predominant condition. Hallmark symptoms include the presence of chronic or recurrent abdominal pain associated with altered bowel habits without underlying structural abnormalities (1–4). IBS has been subdivided on the basis of predominant bowel habits into diarrhea-predominant (IBS-D), constipation-predominant (IBS-C), or a mix of diarrhea and constipation (IBS-M) subtypes (3). IBS can coexist with other GI disorders including gastroesophageal reflux disease and functional dyspepsia, as well as somatic syndromes including fibromyalgia, interstitial cystitis, migraine headaches, and psychologic disorders (5). Due to its high prevalence, recurrent nature of symptoms and a negative impact on health-related quality of life (6), IBS is associated with substantial cost to patients, the health care system, and society (7).

IBS is considered to be a multi-factorial disorder, however, its pathophysiology is not completely understood. IBS and other functional GI disorders have more recently been redefined by experts as “disorders of gut-brain interactions (DGBI) classified by GI symptoms related to any combination of the following: motility disturbance, visceral hypersensitivity, altered mucosal and immune function, altered gut microbiota, and altered central nervous system (CNS) processing” (4). The presence of emotional and psychological factors and food intolerance contribute to the clinical presentation and can exacerbate IBS symptoms (8, 9).

Studies have shown that genetic factors have a modest effect in IBS (10). In addition, there is increasing evidence of a strong influence of environmental factors such as stress in its pathogenesis. A number of studies have found that IBS patients have a higher prevalence of stressful events including early adverse life events (EALs), or traumatic experiences during childhood, as well as current stressful life events in adulthood (11–13). The mechanisms underlying long-term effects of stress and EALs may result from epigenetic programing (14). Epigenetic changes refer to molecular alterations that potentially lead to altered gene expression resulting in a change in phenotype in absence of alteration in the underlying gene sequence.

In this review, we summarize the genetic factors associated with IBS and describe the role of epigenetic factors including DNA methylation and histone modifications as links between genes and environmental factors (e.g., stress) in the etiopathology of IBS. We review the current knowledge of epigenetic modifications associated with IBS in patients as well as in early life stress animal models of IBS, and those associated with IBS endophenotypes (defined as intermediate phenotypes of subclinical traits) including stress and hypothalamic–pituitary–adrenal (HPA) axis function, visceral hypersensitivity and abdominal pain, and GI motility. Further, we briefly outline the role of other epigenetic factors including non-coding RNAs (long non-coding RNAs [lncRNAs] and microRNAs [miRNAs]) in IBS. Finally, we will present a schematic model of our current understanding of factors associated with IBS pathogenesis. A better understanding of the epigenetic mechanisms in IBS can open new avenues for the identification of novel therapeutic targets.

## Genetic Changes Associated With IBS

### Familial Aggregation and Twin Studies in IBS

IBS is often associated with familial clustering in which patients report a family history of IBS (15–17). However, the strength of the genetic association varies between studies. One study reported familial aggregation in IBS but found no evidence of association in spouses, suggesting either a possible genetic etiology or an exposure to a shared household environmental factor early in life as an underlying cause of IBS (18). Additional evidence in favor of both a genetic and environmental etiology of IBS comes from twin studies. Twin studies by Morris-Yates et al. (19) and Svedberg et al. (20) provided evidence for genetic basis of IBS in Australian and Swedish populations. In two large studies on 281 twin pairs in the United States (21) and 3334 twin pairs in Norway (22), Levy et al. and Bengtson et al. showed a higher concordance rate among monozygotic twins than in dizygotic twins for IBS. However, one study by Mohammed et al. (23), failed to replicate the differences in the concordance rates between the monozygotic and dizygotic twin groups. Interestingly, Levy et al. also reported that the presence of IBS in the mother was a strong predictor of having IBS. The proportion of twins who had mothers with IBS was 15.2% which was significantly higher than the 6.7% of twins with IBS who had a co-twin with IBS. Since dizygotic twins share about the same number of genes with each other as each twin shares with their mother, this study suggested that in addition to heredity, social learning, and behavior may contribute to the development of IBS (24).

### Candidate Gene Studies in IBS

IBS has been associated with genetic variants in a number of candidate genes. Genes associated with IBS in various studies are listed in **Table 1**. These include single nucleotide polymorphisms (SNPs) in genes related to signaling systems important in the control of gut motility or sensation in IBS, which includes serotoninergic (5-HT) system including tryptophan hydroxylase (TPH), serotonin reuptake transporter (SERT), a, cholecystokinin (CCK), voltage-gated sodium channels (Nav), Catechol-O-methyltransferase (COMT), cannabinoids, and ion channels, such as transient receptor potential (TRP) channels (TRPV1). Immune related SNPs have been of particular interest in IBS based on accumulating evidence showing immune activation in IBS (25). However, findings have been variable across studies and association of genes such as tumor necrosis factor (TNFα) and IL-10 have not been consistent (26). A recent meta-analysis, which included 12 published case-control studies found no significant association with IBS with polymorphisms in genes such as IL-4, IL-6, IL-8, IL-10, TNFA, IL-1R1, and IL-23R. However, SNP rs4263839 which encodes for TNFSF15 was only moderately associated with IBS, in particular with IBS-C (25). Candidate gene association studies in IBS have been comprehensively reviewed by Cheung et al. (27), Camilleri (28), and Gazouli et al. (29).

**Table 1:** Genetic changes associated with irritable bowel syndrome (IBS).

**Function**	**Gene**	**Polymorphism**	**Endophenotype**	**PMID**
**Neurotransmission**				
Serotonin biosynthesis	Tryptophan hydroxylase (*TPH1* and *TPH2* isoforms)	rs4537731, rs211105, rs4570625	IBS-D, IBS-C	21073637, 24060757
Serotonin reuptake; Seretonin receptors	Serotonin reuptake transporter (*SERT* or *SLC6A4*); 5-HT receptor 3A (*HTR3A*)	5-HT transporter linked promoter region (5-HTT LPR) deletion; rs25531; rs1062613	IBS-C, IBS; IBS-D, symptom severity and anxiety	12135035, 15361494, 17040410, 17564628, 17074108, 17241856, 18511740, 19426812, 19125330; 19125330, 24069428, 24512255, 21420406.
Adrenergic receptors, Catecholamine metabolism	Adrenergic receptors alpha (*ADR2A*, *ADR2C*, *ADRA1D*), Catechol-o-methyl transferase (*COMT*)	alpha(2C) Del 322–325; alpha(2A) −1291; rs1556832, val158met	IBS-C, severity, alterations in brain regions, IBS	19833115, 26288143
Neuropeptide receptors	Neuropeptide S receptor1 (*NPSR1*)	rs2609234, rs6972158, rs1379928, rs1379928	colonic transit, pain and gas	21437260
Cannabinoid mechanisms	Cannabinoid receptor1, (*CNR1/CB1*), Fatty acid amide hydrolase (*FAAH*), Corticotropin-releasing hormone binding protein (*CRHBP*)	AAT repeat frequency, rs806378 C385A, rs10474485	IBS, abdominal pain, IBS-D, colonic motility, transit time, emotional abnormalities	19732772
**Barrier function, Immune and Inflammatory Mediators**				
Barrier function, adhesion	Toll-like receptor 9 (*TLR9*), Cadherein 1 (*CDH1*)	rs5743836	PI-IBS, epithelial cell barrier function	20044998
Cytokines	Interleukin (*IL*)-*6*, *IL-10*, Tumor necrosis factor-alpha (*TNFα*), *IL-8*, *TNFSF15*	rs1800870, rs1800872, rs6478108, rs6478109, rs7848647, rs4263839	PI-IBS, IBS, IBS-D, innate immune response	20044998; 22837345
**Ion Channels and Bile acids**				
	Voltage-gated sodium channel NaV 1.5 (*SCN5A*), G protein-coupled bile acid receptor 1 (*GPBAR1*), Klotho Beta (*KLB*)	rs11554825, rs17618244	IBS, colonic transit, fecal bile acid	20044998, 21752155, 16279907, 23595519, 12477767, 15765388, 20337945, 22158028, 24409078, 22684480, 21636646, 25824902

**Table 1** shows genetic changes associated with IBS and IBS endophenotypes. PMID, PubMed ID; IBS-D, IBS diarrhea subtype; IBS-C, IBS constipation subtype.

Despite these genetic associations, it is not entirely unexpected that the effects of an individual polymorphism on the overall phenotype are modest because IBS is a complex, multifactorial condition. Moreover, the development of disease likely involves more than the presence of just a moderately associated common variant. While SNPs of these genes alone may not be sufficient to cause IBS or other complex chronic pain conditions, they may interact with other genes and environmental factors including EALs and contribute to the disease etiology. Therefore, an alternative approach has been to evaluate an association of gene variants with specific IBS subtypes (IBS-D, IBS-C, and IBS-M) as well as endophenotypes. For example, we found that the SNP rs1556832 in the catecholaminergic gene, adrenoceptor alpha 1D (ADRA1D), was associated with IBS symptom severity and morphological changes in brain regions that modulate sensory processing (30). In another study, we demonstrated that the presence of IBS was significantly associated with SNPs in corticotropin releasing hormone receptor 1 (*CRH-R1*) gene. These SNPs were associated with increased GI symptom-related anxiety and acoustic startle response to threat in IBS patients, suggesting that that CRH-R1 is involved in altered stress responsiveness in IBS (30).

### Genome Wide Association Studies (GWAS) in IBS

Considering the challenges of identifying individual risk alleles in case-control studies and the difficulty of defining significant gene association with IBS, a GWAS using large samples has been proposed as an alternative approach in an attempt to increase sample size and homogeneity. Ek et al. reported a GWAS study in IBS comprising of 534 IBS patients and 4,932 healthy controls, followed by six independent clinical case-control replication studies from different countries (31) where they identified variants in *KDLER2* and *GRIP2IP* (chromosome 7p22.1) genes to be associated with IBS. *KDLER2* codes for a family of integral membrane protein with seven transmembrane domains involved in intracellular signaling of bacterial toxins (32), potentially relevant to the role of microbiota in IBS. The *GRID2IP* gene encodes for a protein (delphilin) expressed on fiber-Purkinje cell synapses in the brain involved in glutamatergic neurotransmission, potentially relevant to pain signaling (31, 33). Another GWAS with a smaller sample size (172 IBS cases and 1,398 controls) conducted in an Australian cohort found an association of protocadherin 15 (*PCDH15*) gene, encoding an integral membrane protein that mediates calcium-dependent cell–cell adhesion (P~9 × 10−9).

GWAS studies have also evaluated other SNP associations in IBS. TNFSF15 was found to be only nominally significant in the GWAS study, contrasting with prior reports as mentioned previously. Similar nominal associations were detected for other genes such as Cell Division Cycle 42 (*CDC42*), Neurexophilin 1 (*NXPH1*) (34), 5-HT Receptor 3E (*HTR3E*) (35), Klothoβ (*KLB*) (36) and Sodium Voltage-Gated Channel Alpha Subunit 5 (*SCN5A*). Interestingly, *SCN5A* encodes the α-subunit of the voltage-gated sodium channel NaV1.5. About 2% of patients with IBS were found to carry mutations in *SCN5A*, most of which were loss-of-function mutations that disrupted NaV1.5 channel function (37). Additionally, in a GWAS study on self-reported IBS patients and controls, Bonfiglio et al. identified variants at 9q31.2 locus that were associated with IBS in women suggesting a role for sex hormones in IBS (38). However, most genes associated with IBS thus far represent non-validated findings and therefore their role in IBS needs to be cautiously interpreted. Moreover, such discrepancies are believed to arise from multifactorial nature of the disease, phenotype heterogeneity (including variability in endophenotypes) and/or sample sizes, among others.

Additionally, the mechanisms involved in pain sensitization and altered motility are likely multifactorial as demonstrated in multiple clinical and animal studies in the past decades (39). These functional alterations are mediated through cellular and molecular changes mediated by genetic and epigenetic alterations (40) detailed in the following sections. At the CNS level, proposed mechanisms include plasticity of the endogenous pain modulation system and structural changes in the brain (41, 42). An important step towards understanding the complex pathogenesis of IBS lies in the ability to discover the interface between genetic pathways and epigenetic regulation mediated by gene-environment interaction at peripheral (gut) and central (CNS) levels.

## Stress: An Environmental Trigger for IBS

IBS is associated with various environmental factors including chronic stress in early life and/or adulthood, diet (43–45), and gastrointestinal infections (46, 47). Chronic stress can increase an individual’s vulnerability to developing IBS and/or can trigger or exacerbate the symptoms of IBS (48, 49). Stress is the body’s reaction to a physical or psychological stimulus that disturbs the homeostasis of an organism. Stress has wide-spread effects on gut physiology, including changes in intestinal motility, mucosal transport, and gut barrier function leading to changes in permeability, and visceral perception. The biological effects of stress are mediated by the sympathetic nervous system and corticotropin releasing factor (CRF)/HPA axis pathways. Glucocorticoids, which are major effector molecules of the HPA axis, bind to their intracellular receptors and regulate the physiological adaptations to stress (50, 51). Glucocorticoids including cortisol/corticosterone initiates negative feedback control *via* binding to glucocorticoid receptors (GR) and mineralocorticoid receptors (MR) in brain regions including hippocampus, paraventricular nucleus (PVN), and anterior pituitary gland (52). However, in response to chronic and uncontrollable stressors, maladaptive changes can be elicited resulting in malfunctioning of stress systems affecting the brain structure and function (53, 54).

### Stress-Induced Visceral Hypersensitivity and Motility Abnormalities in IBS

Many studies support an important role for stress in the IBS pathophysiology and symptoms (8). The stress-induced activation or augmentation of the CRF and HPA axis systems has been associated with visceral hypersensitivity, an important feature of IBS, in animal models (55–58). IBS patients have a greater reactivity to stress compared to healthy subjects, as manifested by a dysregulated HPA axis response, enhanced visceral perception and gut motility, among other findings (59–61). IBS has been associated with increased prevalence of EALs and a growing body of evidence from both animal and human studies supports the hypothesis that chronic stress, including EALs, represent an important mechanism leading to changes in glucocorticoid receptor (GR) expression, thereby increasing responsiveness of the HPA axis (62). The HPA axis response is regulated by a negative feedback though binding of cortisol to GRs at multiple levels including the hypothalamus and hippocampus. Impairment of this negative feedback mechanism can lead to a dysregulation of the HPA axis, specifically an enhanced HPA axis response due to reduced negative feedback from reduced expression of GRs. The importance of an early life and adulthood stress on this IBS phenotype was demonstrated in the maternal separation (MS) animal model, where pups that were maternally separated in early life and later subjected to psychologic stress as an adult displayed post‐stress visceral hypersensitivity, increased corticosterone levels, and reduced expression of GRs in the hippocampus (11, 12). Additionally, stress-induced visceral hyperalgesia has been investigated in repeated water avoidance stress (WAS), a validated rat model of psychological stress that demonstrates many human IBS-like traits. A knockdown of GRs has also been shown to increase visceromotor response to colonic distention in animal models (63). Additionally, a neonatal inflammation rat model suggested a role for inflammatory insult in early life, which upregulates vasoactive intestinal peptide (Vip) in the colon muscularis externa contributing to altered motility and diarrhea-like symptoms as seen in IBS-D patients (64–66).

We found that GR expression was decreased in peripheral blood mononuclear cells (PBMCs) in IBS patients in comparison to healthy controls and that GR expression levels negatively correlated with pituitary responsiveness (ACTH levels) to CRF stimulation (67). That is, reduced GR expression was associated with an enhanced HPA axis response. HPA axis function was assessed in PBMCs because they are accessible and feasible to study. Although GRs regulate HPA axis *via* negative feedback in the CNS, changes in GRs on PBMCs have been reported in psychiatric diseases, including changes in the number and sensitivity of GRs (68, 69) and GR promoter methylation status and mRNA expression (67, 70–72). Furthermore, the transcriptome of peripheral blood has been shown to share >80% homology with genes expressed in the brain, heart, liver, spleen, colon, kidney, prostate, and stomach, and that there is a broad movement of leukocyte subsets to and from the gut at steady state, suggesting that PBMCs can reflect the molecular events at the central and peripheral locations (73).

### Stress, Intestinal Epithelial Barrier Function and Immune System

Various animal models representing different stress paradigms (e.g. restraint stress, WAS, neonatal MS, etc.) as well as studies in human subjects have demonstrated an impairment in mucosal barrier function, the enteric nervous system (ENS), and immune system (74–76). These stress-induced changes result in alterations in GI functions including increased intestinal permeability, altered ion transport and hypersecretion, and mucus secretion and are mediated by neuro-immune mechanisms including the CRF system, which consists of CRF, urocortins 1–3 (Ucn) and their receptors CRF-1R and CRF-2R (77, 78). Barrier dysfunction may also occur early in IBS and is hypothesized to contribute to low-grade intestinal immune activation and increased visceral perception (79), specifically in IBS-D patients (80, 81) and post-infection IBS (PI-IBS) (82). Additionally, an increase in paracellular permeability has been correlated with the magnitude of visceral pain in IBS-D patients (81). Furthermore, an exaggerated response to CRH infusion in IBS patients was associated with an increase in cytokine levels suggesting a correlation between stress and increased cytokine levels (83). This is hypothesized to be mediated by glucocorticoid-related epigenetic changes leading to inadequate suppression of proinflammatory cytokines (40). It conceivable that this contributes, at least in part, to the higher plasma levels of cytokines reported in some IBS patients (84, 85).

Both local (intestinal) and systemic factors contribute to the altered epithelial barrier function. Recent data indicate that soluble mediators from fecal supernatants and mucosal homogenates of IBS patients affect the epithelial integrity, thereby increasing colonic permeability (86, 87). These studies showed that the impairment of barrier integrity may be mediated *via* the release of various mediators such as serine proteases or histamine. Additionally, molecular alterations such as expression of genes involved in barrier function (88) can mediate permeability changes, which can in turn lead to permeation of bacteria and their products.

## Epigenetic Modifications: A Bridge Between Environment and Genes in IBS

**Figure 1:**
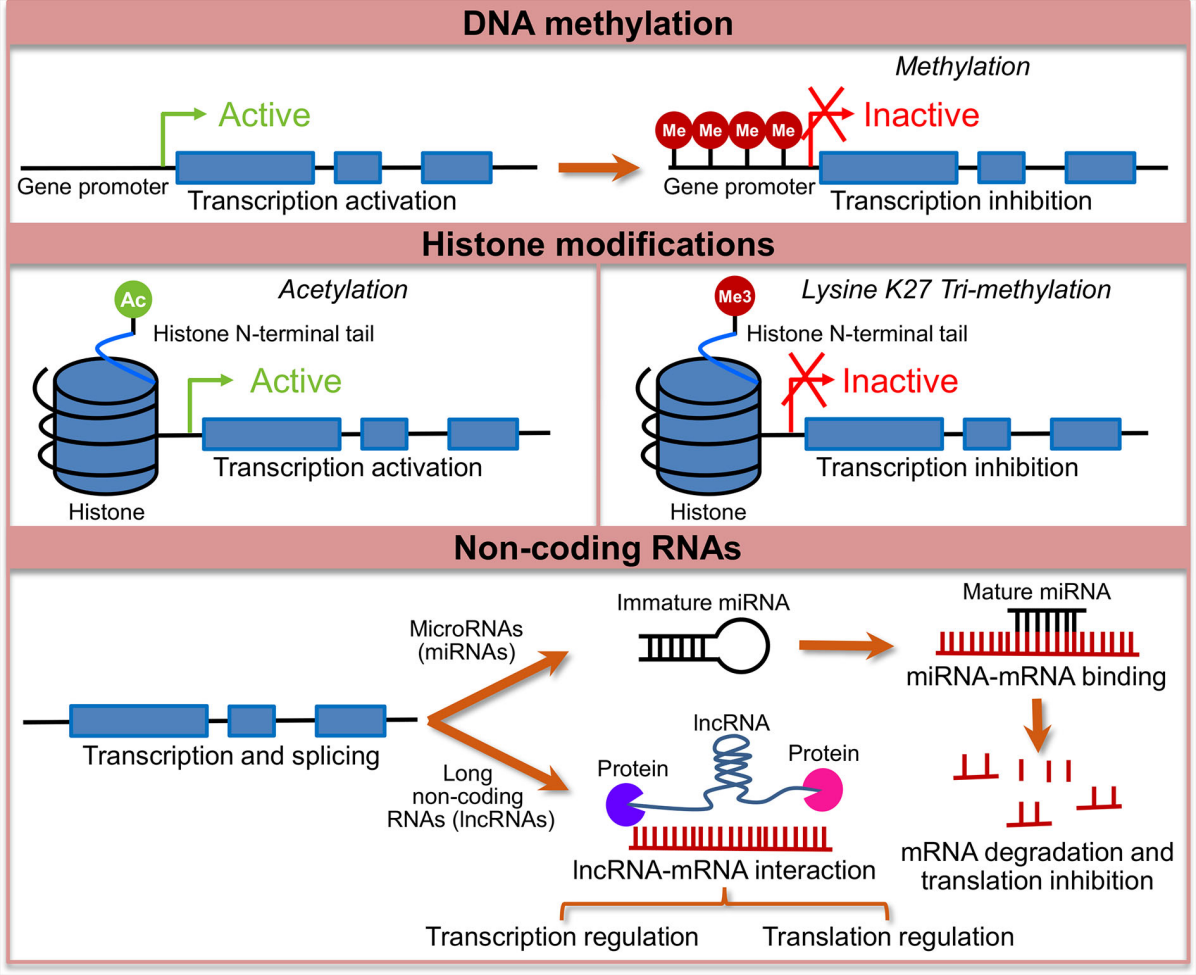
Major epigenetic changes studied in the context of irritable bowel syndrome (IBS). Shows a conceptual model of major epigenetic changes studied in the context of IBS. Lines with blue boxes represent genes with promoter regions. Blue boxes represent exons, lines before exon 1 represents promoter region and the lines between exons represent introns. The top panel shows active transcription in the unmethylated state of the gene, which when methylated (Me) at the promoter region leads to transcription inactivation. Middle panel shows two representative histone modifications, histone acetylation at the N-terminal tail, which is usually associated with activation of transcription and histone methylation, specifically, addition of a tri-methyl group (Me3) at 27^th^ lysine (K) on the N-terminal tail, which is associated with transcription repression. The bottom panel shows mechanism of transcription regulation by non-coding RNAs. MicroRNA genes are transcribed to immature precursor miRNAs that are processed to form mature miRNAs, which bind to miRNAs either leading to mRNA degradation or inhibition of translation. Long non-coding RNAs regulate transcription and translation, and function at the level of chromatin *via* interaction with RNA binding proteins.

Epigenetic mechanisms alter gene expression without alterations of underlying DNA sequence (89) and are key to the normal development, cellular function, and differentiation into specific lineages (90). These mechanisms broadly include DNA methylation, histone modification, and non-coding RNA mediated gene regulation as shown in **Figure 1**. Epigenetic mechanisms play a role in synaptic plasticity, learning, and memory (91), as well as in various neuropsychiatric conditions including depression and pain (92). Long-lasting epigenetic changes have been linked to early stress, childhood trauma or abuse. Epigenetic changes are amenable to exogenous influences and involve complex and dynamic interaction between the DNA sequence, DNA and histone modifications and environmental factors, all of which combine to produce the phenotype, thus providing an important link between environment and phenotype (93).

### DNA Methylation in Animal Models of IBS and IBS Patients

In vertebrates, DNA methylation occurs mostly in the context of CpG dinucleotides by a covalent attachment of a methyl group to the C5 position of cytosine (89). CpG islands (CGIs) are short interspersed DNA sequences (usually 1000 base-pairs) with a high concentration of CpG residues, which are normally non-methylated in contrast to the rest of the genome, which is globally methylated. CGIs typically occur at or near the transcription start site of genes (94) and when a CGI in the promoter region of a gene is methylated, expression of the gene is repressed. The exact mechanism of DNA methylation mediated repression of gene expression has begun to be elucidated in recent years. DNA methylation results in binding of methyl-binding-domain (MBD) proteins, which are associated with large protein complexes that contain histone deacetylases (HDACs) and recruit histone methyl transferases (HMTs) leading to chromatin remodeling (95). Both DNA methylation and the proteins associated with MDBs are being investigated as promising therapeutic targets (96). Additionally, recent studies have demonstrated that methylation of CpG sites in the gene body are positively correlated with gene expression and is a potential therapeutic target in cancer (97). The quantification of DNA methylation in diseased or environmentally impacted cells could provide useful information for detection and treatment of the disease.

DNA methylation changes, in particular, have been studied in various chronic conditions including cancer (98), chronic pain (99), and psychiatric diseases (100). Stress and other environmental factors including EALs, diet and gut microbial metabolites can potentially trigger epigenetic alterations (101, 102). For example, studies have demonstrated that maternal care influences HPA axis function through epigenetic programming of GR (coded by Nuclear Receptor Subfamily 3 Group C Member 1, or *NR3C1*) expression and that environment-induced remodeling of the epigenome, or during chronic stress, can result in long-term changes in gene expression (103–105). **Table 2A** lists the epigenetic modifications reported in association with IBS or animal models of IBS. The role of central epigenetic regulatory mechanisms in stress-induced visceral hypersensitivity has been demonstrated in MS and WAS rat models. While MS animal models mimic the early life stress, WAS simulates both acute and chronic effects of a psychological stressor on colonic sensitivity, which have been extensively reviewed by Greenwood-Van Meerveld et al. (106). Stress-induced visceral hypersensitivity has been associated with an increase in DNA methylation in the GR gene promoter and a decreased expression of the GR gene in the amygdala of WAS rats (107, 108). Additionally, the study identified a decrease in DNA methylation and increased expression of the CRF gene associate with visceral hypersensitivity in the amygdala of the stressed rats. Hong et al. demonstrated that chronic stress increased methylation of genes that regulate visceral pain sensation in the peripheral nervous system of rats. They reported that chronic stress resulted in increased promoter methylation and reduced expression of the *NR3C1* (or GR) gene in L6-S2 dorsal root ganglia (109). In human subjects, DNA methylation in brains of suicide victims with a history of childhood abuse was associated with increased methylation and decreased expression of GR gene compared to suicide victims with no history of childhood maltreatment (104). However, no clear consensus exists regarding DNA methylation of the GR gene in IBS patients.

**Table 2A:** Epigenetic changes associated with irritable bowel syndrome (IBS).

Functional category	Gene	Sample	IBS vs controls	Phenotype	PMID
**DNA methylation**					
**Oxidative stress**	Glutathione-S-transferases mu 5 (*GSTM5*)	PBMCs	Hyper-methylated	IBS-D	26670691
**Neuronal genes**	SCO-Spondin (*SSPO*)	PBMCs	Hyper-methylated	IBS; HAD^#^ depression	26670691
	Tubulin polymerization promoting protein (*TPPP*)	PBMCs	Hyper-methylated	IBS-C	26670691
	SSX family member 2 interacting protein (*Ssx2ip*)	Colon of WAS^$^	Hyper-methylated	Visceral hypersensitivity	30106160
	Par-3 family cell polarity regulator (*Pard3*)	Colon of WAS^$^	Hyper-methylated	Visceral hypersensitivity	30106160
	Vinculin (*Vcl*)	Colon of WAS^$^	Hyper-methylated	Visceral hypersensitivity	30106160
	Glucocorticoid receptor (*Nr3c1*)	MS Amygdala/DRG^%^neurons in WAS^$^	Hyper-methylated	Visceral hypersensitivity	25263804; 23084728
	Corticotropin-releasing factor (*Crf*)	Amygdala/DRG neurons in WAS^$^	Hypo-methylated	Visceral hypersensitivity	23084728
	Cannabinoid receptor 1(*Cnr1*)	DRG^%^ neurons in WAS^$^	Hyper-methylated	Visceral hypersensitivity	25263804
**Histone modifications**					
**Neuronal genes**	Transient receptor potential cation channel subfamily V member 1 (*Trpv1*)	DRG^%^ neurons in WAS^$^	Increased histone (H3) acetylation	Visceral hypersensitivity	25263804
	Brain derived neurotrophic factor (*Bdnf*)	Neonatal inflammation	histone acetylene transferase (HAT)	Visceral sensitivity	28439935
**Calcium channels**	*Cacna1c*	Neonatal inflammation	Reduced interaction with histone deacetylase 3 (HDAC3)	Altered motility and diarrhea	23886858

Table 2A shows epigenetic changes, including DNA methylation and histone modifications associated with IBS or IBS models. ^#^HAD, hospital anxiety depression scale; PMID, PubMed ID; ^$^WAS, water avoidance stress; ^%^DRG, dorsal root ganglia; IBS-D, IBS diarrhea subtype; IBS-C, IBS constipation subtype; Hyper-methylation, increased methylation; Hypo-methylation, decreased methylation.

In a genome-wide methylation scan followed by targeted sequencing, we previously demonstrated an association of DNA methylation of several CpG sites in PBMCs in IBS patients compared to healthy controls (110). We reported an increase in DNA methylation in genes including sub-commissural organ (SCO)-Spondin (*SSPO*), glutathione-S-transferases mu 5 (*GSTM5*) and tubulin polymerization promoting protein (*TPPP*) in IBS patients compared to healthy controls. SSPO is associated with neuronal function (111) and has been suggested to play a role in depression and evidence suggests that SCO secretory activity is regulated by the serotonin system, which plays an important role in stress-related pathways and in IBS (112). Additionally, an increased methylation of *GSTM5*, a gene that codes an enzyme that plays an important role in antioxidant defense was associated with decreased gene expression compared to controls. Although a role for oxidative stress and the significance of epigenetic silencing of *GSTM5* in IBS is not known, DNA methylation mediated repression of *GSTM5* gene expression has been shown in other conditions (113). Although larger independent studies may be required to confirm the functional role of the associated genes, these studies highlight the importance of epigenetic changes in IBS. DNA methylation changes in blood cells can provide insights into systemic changes associated with IBS and can serve as important diagnostic and prognostic biomarkers (114).

Epigenetic changes in the gut mucosa can provide important insights into the peripheral mechanisms of IBS. A recent study investigated the genome-wide methylation predominantly in promoter regions of genes, and gene expression in the colon of rat WAS model and suggested an association of Notch signaling and focal adhesion pathways with psychological stress (115). In a recent study that included a relatively large cohort of IBS subjects and healthy controls (n=102 and 36, respectively), we found several DNA methylation changes in PBMCs as well as colonic mucosa that were associated with IBS. There was increased methylation of stress-related genes such as *NR3C1*, *CRHR1*, brain-derived neurotrophic factor (BDNF) in PBMCs and/or colon (116). In the colonic mucosa of IBS patients, we identified distinct clusters of DNA methylation patterns highlighting the heterogeneity in the epigenetic profiles of colonic mucosa of IBS patients. A hyper-methylated cluster was associated with higher symptom severity and abdominal pain compared to clusters with lower methylation levels and included genes such as protocadherins (PCDHs), cadherins (CDHs), VIP, TRPV4, and Guanylate Cyclase 1, Soluble, Beta 3 (GUCY1B3) which were significant after correcting for multiple comparisons. Thus, these studies suggest that DNA methylation changes are important pathophysiologic mechanisms in IBS and should be further evaluated.

### Histone Modifications in Animal Models of IBS

In eukaryotic cells, genes complex with histone and other chromosomal proteins to form a chromatin scaffold. Histone modifications play an important role in regulation of gene expression. The histone tails undergo a variety of covalent modifications, that include lysine acetylation, methylation, ubiquitination, and sumoylation, among others (117) (**Figure 1**). Acetylation and methylation are some of the most studied histone modifications so far. In general, acetylation of core histone tails leads to open chromatin structure to allow transcription and the histone deacetylases (HDACs) oppose the effects of histone acetylases and are predominantly transcriptional repressors (118). Histone methylation is more complex and can occur on a specific lysine or arginine residue. Depending on the residues being methylated and the number of methylation molecules added (each methylated lysine residue can exist in a mono-, di-, or tri-methylated state), histone methylation may be associated with either an active or a silent state of chromatin. For example, H3K27me3 is associated with transcription repression whereas, H3K4me3 is generally associated with active transcription.

Recent studies have highlighted antinociceptive effects of histone acetylation and lysine tri-methylation in inflammatory and neuropathic pain models (119, 120). In the partial sciatic nerve ligation model of neuropathic pain, an increase in the expression levels of monocyte chemotactic protein-3 (MCP3), a pro-inflammatory cytokine was associated with reduced levels of repressive histone methylation, H3K27me3 (121). A role for histone acetylation has been suggested in the pathophysiology of visceral hypersensitivity induced by early-life stress in the MS animal model of IBS (122). Moloney et al. showed that HDAC inhibitor, suberoylanilide hydroxamic acid (SAHA), reversed visceral hypersensitivity, and the effects of stress on fecal pellet output in animal models of early life stress highlighting the importance of histone acetylation in stress-related conditions (123). Hong et al. demonstrated an increased expression of histone acetyltransferase EP300, which induced acetylation of histone H3 of promoter of nociceptive endovanilloid TRPV1 gene in the chronic WAS model of IBS. Moreover, they demonstrated that siRNA mediated knockdown of EP300 prevented visceral hyperalgesia (124).

Animal models suggest that neonatal inflammation may contribute to altered gut motility *via* histone modification. In rats subjected to neonatal inflammation, Vip levels increased, which reduced the interaction of histone deacetylase 3 (HDAC3) with α1C-subunit of Cav1.2b channel (Cacna1c or α1C1b). This resulted in increased acetylation of histone H3 lysine 9 (H3K9) in the promoter region inducing the transcription of α1C1b which may result in gut dysmotility and diarrhea (65). Similarly, neonatal immune challenge led to an upregulation of tyrosine hydroxylase in the locus coeruleus, mediated by epigenetic programming (125). The study showed a cascade of events involving upregulation of norepinephrine, activation of adrenergic receptors, and involvement of enhanced pCREB binding to the cAMP response element, which resulted in recruitment of histone acetylene transferase (HAT) to the brain derived neurotrophic factor (BDNF) gene. This led to an enhanced expression of the BDNF and aggravated visceromotor response to colorectal distension.

### MicroRNA in Animal Models of IBS and IBS Patients

MiRNAs are endogenous noncoding RNAs of small size (18–25 nucleotides) that have been characterized as important gene expression regulators *via* binding through complementary sequence homology to the 3′-untranslated region (UTR) of target mRNAs thereby causing repression of translation or mRNA degradation (126) (**Figure 1**). Involvement of miRNA in cancer is well established and emerging research indicates a role of miRNA in the regulation of genes that play a role in nociceptive circuits (127). It has been suggested that miRNAs interacting with nervous and immune systems may act as “master switches” regulating a network of genes orchestrating the pain response and may be targeted for therapeutic purposes contrasting with the current strategy focusing on single targets (127). This approach is highly relevant to the GI tract where neuroimmune interactions are key contributors to the control of GI functions.

Recent translational studies in IBS have identified several miRNAs (**Table 2B**) that appear to be important in regulating the expression of genes involved in visceral pain response or intestinal permeability. In a study conducted in two independent cohorts of IBS-D women in in the UK and Germany, there was an association between the c.*76G>A variant in the 3′UTR of the serotonin receptor 3 subunit gene (*HTR3E*), leading to increased expression of the 5HT3E subunit, and (129). Using luciferase assays, this variation was located in the binding element sequence of miR-510 suggesting a functional implication of the *HTR3E* variation in the ability of miR-510 to regulate its gene expression.

**Table 2B:** Non-coding RNAs associated with irritable bowel syndrome (IBS).

	**Targets**	**Endophenotype**	**Sample**	**miRNA regulation in IBS/model**	**PMID**
**MicroRNAs**					
**miR-510**	5-hydroxytryptamine receptor 3E(HTR3E), PRDX1	IBS-D	Colonic mucosa, and cells	Downregulated	18614545 26787495 31934286
**miR-150 and** **miR-342-3p**	Exploratory	Inflammatory and pain pathways	Whole blood	Upregulated	24768587
**miR-199a**	Transient receptor potential cation channel subfamily V member 1(TRPV1)	IBS-D, visceral pain	Colonic biopsies	Downregulated	25681400
**miR-29a**	Glutamate-ammonia ligase (GLUL), Aquaporin (AQP) 1, AQP3 and AQP8	Intestinal permeability	Colon and duodenum of IBS; colonic epithelial cells of IBS-D rat models	Upregulated	19951903 29156760
**miR-16**	HTR4 CLDN2	Intestinal sensitivity and motility; permeability	Colon of IBS-D; Jejunum of IBS-D	Downregulated	29089619 28082316
**miR-103**	5-hydroxytryptamine receptor 4 (HTR4)	Intestinal sensitivity and motility	Colon of IBS-D	Downregulated	29089619
**miR-125b**	Cingulin (CGN)	Permeability	Jejunum of IBS-D	Downregulated	28082316
**miR-144**	Occluding (OCLN), Zona Occludens1 (ZO1)	Intestinal permeability	Colon of BS-D rat model	Upregulated	29258088
**miR-200a**	Cannabinoid receptor 1(CNR1), Serotonin transporter (SERT)	Visceral hypersensitivity	Colon of IBS-D rat model	Upregulated	30347941
**miR-24**	SERT	Pain and nociception	Epithelial cells of colon and mouse model of IBS	Upgregulated	26631964
**LncRNAs**					
**GHRLOS**	Motilin	Smooth muscle contraction	Colonic mucosa of IBS	Downregulated	Videlock et al. (128)
**XIST**	SERT	Visceral hypersenitivity	Colon of mouse model of IBS	Upregulated	32446903

**Table 2B** shows epigenetic changes including miRNA and long non-coding RNA expression changes associated with IBS or IBS models. IBS-D, IBS diarrhea subtype.

Fourie et al. investigated whether circulating miRNAs are differentially expressed in a small number of IBS patients compared to healthy controls (130). This study found an upregulation of miR-150 and miR-342-3p, which are involved in inflammatory (131) and pain pathways, in IBS patients compared to healthy controls (132). Subsequent studies from Zhou et al, using a miRNA microarray approach, revealed increased expression of miR-29a in blood microvesicles, small bowel and colonic biopsies from IBS-D patients compared to healthy controls, and it was associated with increased intestinal permeability (133). Glutamine synthetase was confirmed as a target of miR-29A and was significantly reduced in the small bowel mucosa in IBS patients suggesting a relationship between miR-29a, glutamine dependent signaling pathways and intestinal permeability in IBS patients. In a randomized placebo-controlled trial, glutamate was shown to safely and effectively reduce IBS symptoms in post-infection IBS-D patients with increased intestinal permeability (134). Subsequently, Zhou et al. showed increased levels of mir-29A/B and reduced expression of NFKB Repressing Factor (*NKRF*) and Claudin 1 (*CLDN1*) genes in intestinal tissue from IBS-D patients as well as TNBS colitis and WAS rat models of IBS (135). Additionally, they showed that miR-199a was significantly decreased in IBS-D patients compared to controls and an upregulation in animal models decreased visceral pain *via* inhibition of TRPV1 signaling.

Subsequently, the role of other miRNAs has been identified in IBS. CGN and CLDN2, associated with barrier function were shown to be the targets of hsa-miR-125b-5p and hsa-miR-16, which were downregulated in jejunal mucosal samples of IBS-D (136). Similarly, occludin (OCLN) and zonula occludens 1 (ZO1/TJP1), which are associated with intestinal permeability, were identified as direct targets of miR-144 in the colon of IBS-D rat models (137). In addition, the role of miRNAs in visceral hyperalgesia has been suggested by altered levels of miRNAs, including miR-200a which targets cannabinoid receptor 1 (CNR1) and serotonin transporter (SERT) (138), miR-214 which targets SERT (139), and miR-16 and miR-103 which target HTR4 (140) in a rat model of IBS-D and human IBS-D colonic epithelial cells.

These studies have led to an increased understanding of the molecular mechanisms underlying some of the endophenotypes of IBS. Thus, they may be explored as diagnostic tools and have potential to form a basis for the therapeutic interventions being proposed in IBS (133). However, further studies examining their exact mechanisms in IBS and that can reproduce previous findings in a larger population are needed. These translational discoveries have prompted growing interest in miRNA-based therapy for IBS, although delivering drugs targeting miRNA to the intestinal tissue currently stands as a major obstacle and is being actively investigated (141).

### Long Non-Coding RNAs in IBS

LncRNAs are transcripts that measure more than 200 nucleotides in length and are processed similar to protein-coding mRNAs (142). Although the functional mechanisms of most lncRNAs are not fully understood, they are known to exhibit diverse functional roles, including the gene regulation by chromatin remodeling, modulation of gene expression, localization, and stability (143) (**Figure 1**). Recently, Videlock et al. investigated the entire colonic mucosal transcriptome and found that a lncRNA, GREHLOS, which regulates the expression of motilin involved in smooth muscle contraction, was downregulated in IBS patients compared to healthy controls (128). Recently, increased expression of a lncRNA, X inactivate-specific transcript (XIST) was associated with decrease SERT transcription and increased visceral hypersensitivity in mouse model of IBS-D. The study suggested a role for XIST in recruiting DNA methyl transferases, DNMT1, DNMT3A, and DNMT3B to reduce SERT transcription *via* promoter methylation.

### Microbiome and Diet as Environmental Factors Mediating Epigenetic Changes in IBS

Recent studies are starting to investigate an interaction of microbiome, diet, and epigenetics defined as “microbiota-nutrient metabolism-epigenetics axis” in complex diseases (144). Evidence suggests that epigenetic events are dynamic and responsive to changing nutrient availability and microbiome (102, 144, 145). Although the role of microbes and their metabolites on epigenetic machinery in the manifestation of IBS symptoms has not been investigated, there is indirect evidence for the role of microbial products involved in epigenetic modifications in eliciting visceral hypersensitivity (146). These interactions may be mediated by metabolites synthesized by commensal bacteria including neurotransmitters or short-chain fatty acids (SCFAs) (147). SCFAs, including butyrate, propionate, and acetate produced by the fermentation of host dietary polysaccharides, have neuroactive properties (148) and may play an important role in the brain-gut microbiome axis in IBS (149). SFCAs have been shown to regulate post-translational modifications of histones by inhibiting histone deacetylases, promoting active chromatin state and thereby promoting transcription (150, 151).

Nutrigenomics, the study of interaction of diet and genomic factors is an emerging topic in the context of IBS (152, 153). The majority of patients with IBS report meal-related symptoms and dietary modifications is an increasing treatment intervention used in IBS. For example, a low FODMAP (fermentable oligo-, di-, and mono-saccharides and polyols) diet has been associated with alleviation of IBS symptoms (154–156). Additionally, nutrition or diet can affect the epigenomic state. The role of diet in regulating epigenetic pathways is highlighted by a recent study, which showed that calorie restriction changes gene expression and DNA methylation profile of subcutaneous adipose tissue (145). It is suggested that diet and microbial metabolites influence the epigenome by impacting the pool of compounds or enzymes involved in epigenetic pathways (102). In particular, dietary components, co-factors, and vitamins including, S-adenosyl methionine (SAM), folate, vitamin B12, vitamin B6, acetyl-CoA have been shown to play a role in regulating histone modifications or DNA methylation levels (144). Therefore, investigating interactions between diet, microbiome and epigenetic factors may be important in understanding the etiology of IBS and developing personalized therapy for IBS.

## Model for Etiopathogenesis of IBS

**Figure 2:**
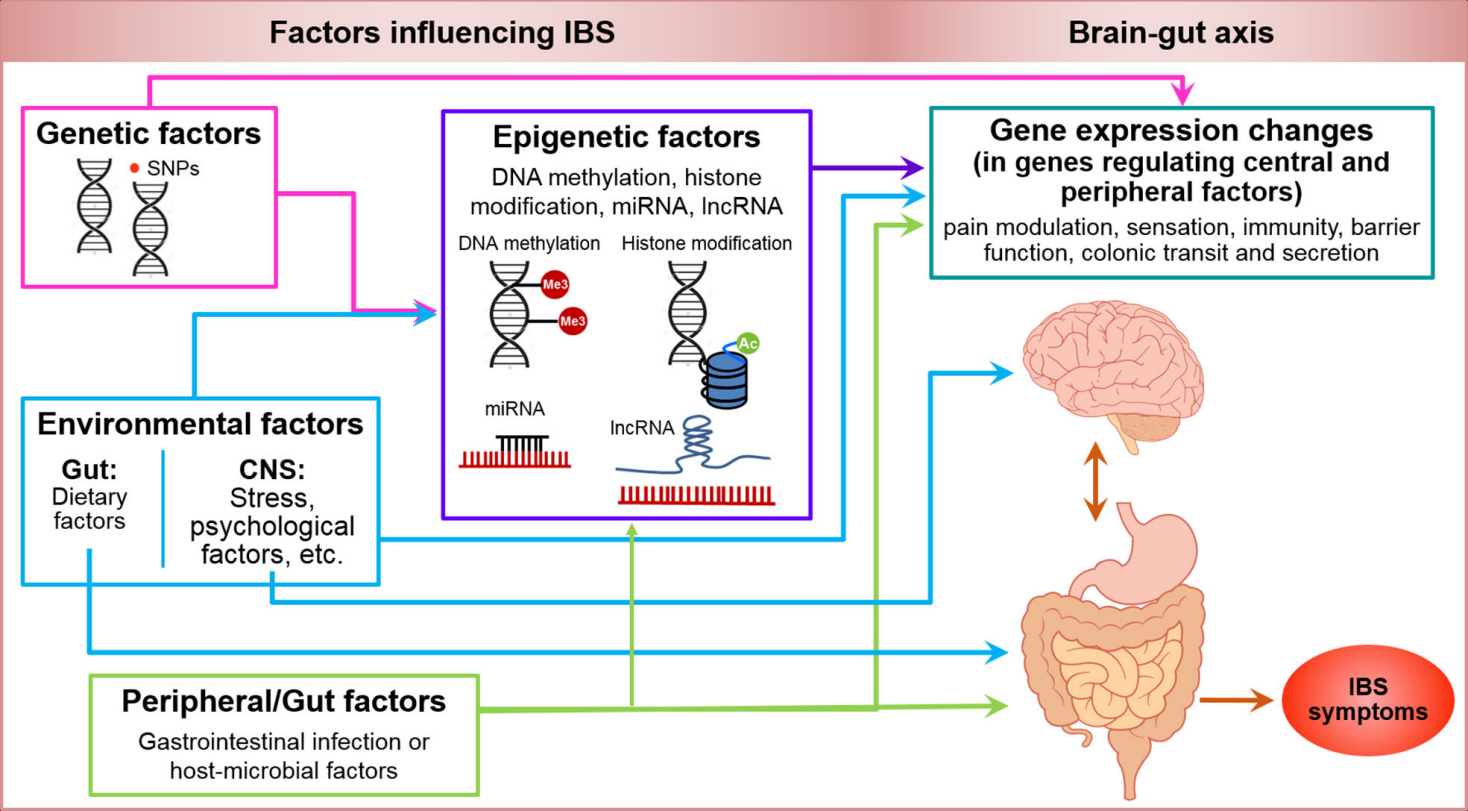
Genetic, epigenetic, environmental and peripheral factors in irritable bowel syndrome. Shows a schematic model of genetic and epigenetic factors influencing IBS. Pink arrows illustrate that genetic factors including SNPs can influence the gene expression either directly or mediated by epigenetic factors including DNA methylation, histone modifications, miRNA and lncRNA expression (purple arrow). Environmental factors including stress and psychological factors at CNS level and dietary factors at gastrointestinal level can induce changes in gene expression mediated by epigenetic or non-genetic/epigenetic factors, and can have a direct influence on CNS and gut function (blue arrows). Peripheral or gut factors including GI infection or other host or microbial factors, can potentially modify the function of genes mediated by epigenetic or non-epigenetic factors, and influence the CNS and gut function (green arrows) such as, pain modulation, sensation, immunity, barrier function, colonic transit and secretion to manifest the symptoms of IBS (orange-red arrow).

Genetic, epigenetic, and other factors associated with IBS have been summarized in a schematic figure (**Figure 2**). IBS is a multifactorial disorder of gut-brain interactions. In addition to stress, diet, and other environmental factors, changes at molecular level including genetic and epigenetic factors may contribute to pain modulation at the CNS level and/or periphery, and affect immune function, oxidative stress, mucosal barrier function, and GI motor and secretory function at the peripheral level in IBS. Moreover, gut microbiota and their metabolites likely contribute to this integrated system and play a major role in the pathogenesis of IBS. Given that IBS is a complex, multifactorial disorder, we propose that epigenomic mechanisms imprint dynamic environmental effects on the fixed genome resulting in alterations in phenotype leading to a disease state. These changes are potentially reversible and can be powerful diagnostic and prognostic markers and therapeutic targets (157, 158).

## Conclusion

Understanding the role of neuroimmune, genetic, epigenetic, and microbial underpinnings in IBS is crucial to understanding the pathophysiology of IBS. The mechanisms of visceral pain and neuro-motor dysfunction, resulting in the symptoms of IBS are influenced by several factors including stress, genetic, epigenetic as well as microbiota. An in-depth investigation of these factors independently, as well as integratively, in a sufficiently large, well-characterized patient and control populations is crucial in understanding the etio-pathology of IBS and in identifying reliable and validated diagnostic biomarkers and therapeutic targets in IBS.

## Author Contributions

SM-J reviewed the literature and wrote the manuscript. LC reviewed the literature, wrote and edited the manuscript and provided the resources.

## Funding

NIH/NIDDK grants P50 DK64539, R21 DK104078, P30 DK 41301, UL1TR000124.

## Conflict of Interest

The authors declare that the research was conducted in the absence of any commercial or financial relationships that could be construed as a potential conflict of interest.

## References

[1] Lovell RM Ford AC Global prevalence of and risk factors for irritable bowel syndrome: a meta-analysis. Clin Gastroenterol Hepatol. (2012) 10:712–721.e4. 10.1016/j.cgh.2012.02.029 22426087

[2] Heitkemper M Jarrett M Bond EF Chang L Impact of sex and gender on irritable bowel syndrome. Biol Res Nurs (2003) 5:56–65. 10.1177/1099800403005001006 12886671

[3] Longstreth GF Thompson WG Chey WD Houghton LA Mearin F Spiller RC Functional bowel disorders. Gastroenterology (2006) 130:1480–91. 10.1053/j.gastro.2005.11.061 16678561

[4] Drossman DA Hasler WL Rome IV-Functional GI Disorders: Disorders of Gut-Brain Interaction. Gastroenterology (2016) 150:1257–61. 10.1053/j.gastro.2016.03.035 27147121

[5] Kim SE Chang L Overlap between functional GI disorders and other functional syndromes: what are the underlying mechanisms? Neurogastroenterol. Motil (2012) 24:895–913. 10.1111/j.1365-2982.2012.01993.x 22863120 PMC3812246

[6] Gralnek IM Hays RD Kilbourne A Naliboff B Mayer EA The impact of irritable bowel syndrome on health-related quality of life. Gastroenterology (2000) 119:654–60. 10.1053/gast.2000.16484 10982758

[7] Canavan C West J Card T Review article: the economic impact of the irritable bowel syndrome. Aliment. Pharmacol Ther (2014) 40:1023–34. 10.1111/apt.12938 25199904

[8] Chang L The role of stress on physiologic responses and clinical symptoms in irritable bowel syndrome. Gastroenterology (2011) 140:761–5. 10.1053/j.gastro.2011.01.032 PMC3039211 21256129

[9] Monsbakken KW Vandvik PO Farup PG Perceived food intolerance in subjects with irritable bowel syndrome– etiology, prevalence and consequences. Eur J Clin Nutr (2006) 60:667–72. 10.1038/sj.ejcn.1602367 16391571

[10] Saito YA Mitra N Mayer EA Genetic approaches to functional gastrointestinal disorders. Gastroenterology (2010) 138:1276–85. 10.1053/j.gastro.2010.02.037 PMC3829382 20176021

[11] Bradford K Shih W Videlock EJ Presson AP Naliboff BD Mayer EA Association between early adverse life events and irritable bowel syndrome. Clin Gastroenterol Hepatol. (2012) 10:385–390.e1–3. 10.1016/j.cgh.2011.12.018 22178460 PMC3311761

[12] Park SH Videlock EJ Shih W Presson AP Mayer EA Chang L Adverse childhood experiences are associated with irritable bowel syndrome and gastrointestinal symptom severity. Neurogastroenterol. Motil (2016) 28:1252–60. 10.1111/nmo.12826 PMC4956522 27061107

[13] Parker CH Naliboff BD Shih W Presson AP Videlock EJ Mayer EA Negative Events During Adulthood Are Associated With Symptom Severity and Altered Stress Response in Patients With Irritable Bowel Syndrome. Clin Gastroenterol Hepatol. (2019) 17:2245–52. 10.1016/j.cgh.2018.12.029 PMC6609507 30616026

[14] Meaney MJ Szyf M Environmental programming of stress responses through DNA methylation: life at the interface between a dynamic environment and a fixed genome. Dialogues Clin Neurosci (2005) 7:103–23. 10.31887/DCNS.2005.7.2/mmeaney PMC3181727 16262207

[15] Whorwell PJ McCallum M Creed FH Roberts CT Non-colonic features of irritable bowel syndrome. Gut (1986) 27:37–40. 10.1136/gut.27.1.37 3949235 PMC1433171

[16] Levy RL Whitehead WE Von Korff MR Feld AD Intergenerational transmission of gastrointestinal illness behavior. Am J Gastroenterol (2000) 95:451–6. 10.1111/j.1572-0241.2000.01766.x 10685749

[17] Locke GR Zinsmeister AR Talley NJ Fett SL Melton LJ Familial association in adults with functional gastrointestinal disorders. Mayo Clin Proc (2000) 75:907–12. 10.4065/75.9.907 10994826

[18] Saito YA Petersen GM Larson JJ Atkinson EJ Fridley BL de Andrade M Familial Aggregation of Irritable Bowel Syndrome: A Family Case–Control Study. Am J Gastroenterol (2010) 105:833–41. 10.1038/ajg.2010.116 PMC2875200 20234344

[19] Morris-Yates A Talley NJ Boyce PM Nandurkar S Andrews G Evidence of a genetic contribution to functional bowel disorder. Am J Gastroenterol (1998) 93:1311–7. 10.1111/j.1572-0241.1998.440_j.x 9707057

[20] Svedberg P Johansson S Wallander M-A Hamelin B Pedersen NL Extra-intestinal manifestations associated with irritable bowel syndrome: a twin study. Aliment. Pharmacol Ther (2002) 16:975–83. 10.1046/j.1365-2036.2002.01254.x 11966507

[21] Levy RL Jones KR Whitehead WE Feld SI Talley NJ Corey LA Irritable bowel syndrome in twins: heredity and social learning both contribute to etiology. Gastroenterology (2001) 121:799–804. 10.1053/gast.2001.27995 11606493

[22] Bengtson M-B Rønning T Vatn MH Harris JR Irritable bowel syndrome in twins: genes and environment. Gut (2006) 55:1754–9. 10.1136/gut.2006.097287 PMC1856463 17008364

[23] Mohammed I Cherkas LF Riley SA Spector TD Trudgill NJ Genetic influences in irritable bowel syndrome: a twin study. Am J Gastroenterol (2005) 100:1340–4. 10.1111/j.1572-0241.2005.41700.x 15929767

[24] Levy RL Jones KR Whitehead WE Feld SI Talley NJ Corey LA Irritable bowel syndrome in twins: heredity and social learning both contribute to etiology. Gastroenterology (2001) 121:799–804. 10.1053/gast.2001.27995 11606493

[25] Czogalla B Schmitteckert S Houghton LA Sayuk GS Camilleri M Olivo-Diaz A A meta-analysis of immunogenetic Case-Control Association Studies in irritable bowel syndrome. Neurogastroenterol. Motil (2015) 27:717–27. 10.1111/nmo.12548 25824902

[26] Bashashati M Rezaei N Shafieyoun A McKernan DP Chang L Öhman L Cytokine imbalance in irritable bowel syndrome: a systematic review and meta-analysis. Neurogastroenterol. Motil (2014) 26:1036–48. 10.1111/nmo.12358 24796536

[27] Cheung CKY Wu JCY Genetic polymorphism in pathogenesis of irritable bowel syndrome. World J Gastroenterol (2014) 20:17693–8. 10.3748/wjg.v20.i47.17693 PMC4273120 25548468

[28] Camilleri M Genetics of Human Gastrointestinal Sensation. Neurogastroenterol. Motil (2013) 25:458–66. 10.1111/nmo.12132 PMC3656127 23594334

[29] Gazouli M Wouters MM Kapur-Pojskić L Bengtson M-B Friedman E Nikčević G Lessons learned — resolving the enigma of genetic factors in IBS. Nat Rev Gastroenterol. Hepatol. (2016) 13:77–87. 10.1038/nrgastro.2015.206 26726033

[30] Orand A Gupta A Shih W Presson AP Hammer C Niesler B Catecholaminergic Gene Polymorphisms Are Associated with GI Symptoms and Morphological Brain Changes in Irritable Bowel Syndrome. PLoS One (2015) 10:e0135910. 10.1371/journal.pone.0135910 26288143 PMC4546052

[31] Ek WE Reznichenko A Ripke S Niesler B Zucchelli M Rivera NV Exploring the genetics of irritable bowel syndrome: a GWA study in the general population and replication in multinational case-control cohorts. Gut (2015) 64:1774–82. 10.1136/gutjnl-2014-307997 25248455

[32] Kreitman RJ Pastan I Importance of the glutamate residue of KDEL in increasing the cytotoxicity of Pseudomonas exotoxin derivatives and for increased binding to the KDEL receptor. Biochem J (1995) 307(Pt 1):29–37. 10.1042/bj3070029 7717988 PMC1136741

[33] Miyagi Y Yamashita T Fukaya M Sonoda T Okuno T Yamada K Delphilin: a novel PDZ and formin homology domain-containing protein that synaptically colocalizes and interacts with glutamate receptor delta 2 subunit. J Neurosci (2002) 22:803–14. 10.1523/JNEUROSCI.22-03-00803.2002 PMC6758529 11826110

[34] Wouters MM Lambrechts D Knapp M Cleynen I Whorwell P Agréus L Genetic variants in CDC42 and NXPH1 as susceptibility factors for constipation and diarrhoea predominant irritable bowel syndrome. Gut (2014) 63:1103–11. 10.1136/gutjnl-2013-304570 24041540

[35] Gu Q-Y Zhang J Feng Y-C Dai G-R Du W-P Association of genetic polymorphisms in HTR3A and HTR3E with diarrhea predominant irritable bowel syndrome. Int J Clin Exp Med (2015) 8:4581–5. PMC4443222 26064388

[36] Wong BS Camilleri M Carlson PJ Guicciardi ME Burton D McKinzie S Gores GJ. A Klothoβ variant mediates protein stability and associates with colon transit in irritable bowel syndrome with diarrhea. Gastroenterology (2011) 140:1934–42. 10.1053/j.gastro.2011.02.063 PMC3109206 21396369

[37] Beyder A Mazzone A Strege PR Tester DJ Saito YA Bernard CE Loss-of-function of the voltage-gated sodium channel NaV1.5 (channelopathies) in patients with irritable bowel syndrome. Gastroenterology (2014) 146:1659–68. 10.1053/j.gastro.2014.02.054 PMC4096335 24613995

[38] Bonfiglio F Zheng T Garcia-Etxebarria K Hadizadeh F Bujanda L Bresso F Female-Specific Association Between Variants on Chromosome 9 and Self-Reported Diagnosis of Irritable Bowel Syndrome. Gastroenterology (2018) 155:168–79. 10.1053/j.gastro.2018.03.064 PMC6035117 29626450

[39] Lee YJ Park KS Irritable bowel syndrome: emerging paradigm in pathophysiology. World J Gastroenterol (2014) 20:2456–69. 10.3748/wjg.v20.i10.2456 PMC3949256 24627583

[40] Dinan TG Cryan J Shanahan F Keeling PWN Quigley EMM IBS: An epigenetic perspective. Nat Rev Gastroenterol Hepatol. (2010) 7:465–71. 10.1038/nrgastro.2010.99 20585338

[41] Mayer EA Tillisch K The brain-gut axis in abdominal pain syndromes. Annu Rev Med (2011) 62:381–96. 10.1146/annurev-med-012309-103958 PMC3817711 21090962

[42] Seminowicz DA Labus JS Bueller JA Tillisch K Naliboff BD Bushnell MC Regional gray matter density changes in brains of patients with irritable bowel syndrome. Gastroenterology (2010) 139:48–57.e2. 10.1053/j.gastro.2010.03.049 20347816 PMC2902717

[43] Singh R Salem A Nanavati J Mullin GE The Role of Diet in the Treatment of Irritable Bowel Syndrome: A Systematic Review. Gastroenterol Clin North Am (2018) 47:107–37. 10.1016/j.gtc.2017.10.003 29413008

[44] Hayes PA Fraher MH Quigley EMM Irritable bowel syndrome: the role of food in pathogenesis and management. Gastroenterol Hepatol. (N. Y.) (2014) 10:164–74. PMC4014048 24829543

[45] Dimidi E Rossi M Whelan K Irritable bowel syndrome and diet: where are we in 2018? Curr Opin Clin Nutr Metab Care (2017) 20:456–63. 10.1097/MCO.0000000000000416 28872467

[46] Koloski NA Jones M Weltman M Kalantar J Bone C Gowryshankar A Identification of early environmental risk factors for irritable bowel syndrome and dyspepsia. Neurogastroenterol. Motil (2015) 27:1317–25. 10.1111/nmo.12626 26202154

[47] Barbara G Grover M Bercik P Corsetti M Ghoshal UC Ohman L Rome Foundation Working Team Report on Post-Infection Irritable Bowel Syndrome. Gastroenterology (2019) 156:46–58.e7. 10.1053/j.gastro.2018.07.011 30009817 PMC6309514

[48] Lackner JM Brasel AM Quigley BM Keefer L Krasner SS Powell C The ties that bind: perceived social support, stress, and IBS in severely affected patients. Neurogastroenterol. Motil (2010) 22:893–900. 10.1111/j.1365-2982.2010.01516.x 20465594 PMC5052070

[49] Bennett EJ Tennant CC Piesse C Badcock CA Kellow JE Level of chronic life stress predicts clinical outcome in irritable bowel syndrome. Gut (1998) 43:256–61. 10.1136/gut.43.2.256 PMC1727204 10189854

[50] Munck A Guyre PM Holbrook NJ Physiological functions of glucocorticoids in stress and their relation to pharmacological actions. Endocr Rev (1984) 5:25–44. 10.1210/edrv-5-1-25 6368214

[51] Bamberger CM Schulte HM Chrousos GP Molecular determinants of glucocorticoid receptor function and tissue sensitivity to glucocorticoids. Endocr Rev (1996) 17:245–61. 10.1210/edrv-17-3-245 8771358

[52] Herman JP Cullinan WE Neurocircuitry of stress: central control of the hypothalamo-pituitary-adrenocortical axis. Trends Neurosci (1997) 20:78–84. 10.1016/s0166-2236(96)10069-2 9023876

[53] Nutt DJ Malizia AL Structural and functional brain changes in posttraumatic stress disorder. J Clin Psychiatry (2004) 65 Suppl 1:11–7. 14728092

[54] Lupien SJ McEwen BS Gunnar MR Heim C Effects of stress throughout the lifespan on the brain, behaviour and cognition. Nat Rev Neurosci (2009) 10:434–45. 10.1038/nrn2639 19401723

[55] Venkova K Johnson AC Myers B Greenwood-Van Meerveld B Exposure of the amygdala to elevated levels of corticosterone alters colonic motility in response to acute psychological stress. Neuropharmacology (2010) 58:1161–7. 10.1016/j.neuropharm.2010.02.012 20170666

[56] Greenwood-Van Meerveld B Moloney RD Johnson AC Vicario M Mechanisms of Stress-Induced Visceral Pain: Implications in Irritable Bowel Syndrome. J Neuroendocrinol. (2016) 28. 10.1111/jne.12361 26749172

[57] Taché Y Million M Role of Corticotropin-releasing Factor Signaling in Stress-related Alterations of Colonic Motility and Hyperalgesia. J Neurogastroenterol. Motil (2015) 21:8–24. 10.5056/jnm14162 25611064 PMC4288101

[58] Larauche M Moussaoui N Biraud M Bae WK Duboc H Million M Brain corticotropin-releasing factor signaling: Involvement in acute stress-induced visceral analgesia in male rats. Neurogastroenterol. Motil (2019) 31:e13489. 10.1111/nmo.13489 30298965 PMC6347489

[59] Welgan P Meshkinpour H Beeler M Effect of anger on colon motor and myoelectric activity in irritable bowel syndrome. Gastroenterology (1988) 94:1150–6. 10.1016/0016-5085(88)90006-6 3350284

[60] Posserud I Agerforz P Ekman R Björnsson ES Abrahamsson H Simrén M Altered visceral perceptual and neuroendocrine response in patients with irritable bowel syndrome during mental stress. Gut (2004) 53:1102–8. 10.1136/gut.2003.017962 PMC1774150 15247175

[61] Dickhaus B Mayer EA Firooz N Stains J Conde F Olivas TI Irritable bowel syndrome patients show enhanced modulation of visceral perception by auditory stress. Am J Gastroenterol (2003) 98:135–43. 10.1111/j.1572-0241.2003.07156.x 12526949

[62] Videlock EJ Adeyemo M Licudine A Hirano M Ohning G Mayer M Childhood trauma is associated with hypothalamic-pituitary-adrenal axis responsiveness in irritable bowel syndrome. Gastroenterology (2009) 137:1954–62. 10.1053/j.gastro.2009.08.058 PMC2789911 19737564

[63] Winston JH Xu G-Y Sarna SK Adrenergic stimulation mediates visceral hypersensitivity to colorectal distension following heterotypic chronic stress. Gastroenterology (2010) 138:294–304.e3. 10.1053/j.gastro.2009.09.054 19800336 PMC2813397

[64] Sarna SK Colonic Motility: From Bench Side to Bedside (2010). San Rafael (CA: Morgan & Claypool Life Sciences Available at: http://www.ncbi.nlm.nih.gov/books/NBK53477/ (Accessed July 13, 2020). 21452445

[65] Li Q Winston JH Sarna SK Developmental origins of colon smooth muscle dysfunction in IBS-like rats. Am J Physiol Gastrointest. Liver Physiol (2013) 305:G503–512. 10.1152/ajpgi.00160.2013 PMC3798719 23886858

[66] Choudhury BK Shi X-Z Sarna SK Gene plasticity in colonic circular smooth muscle cells underlies motility dysfunction in a model of postinfective IBS. Am J Physiol Gastrointest. Liver Physiol (2009) 296:G632–642. 10.1152/ajpgi.90673.2008 PMC2660181 19136376

[67] Videlock EJ Shih W Adeyemo M Mahurkar-Joshi S Presson AP Polytarchou C The effect of sex and irritable bowel syndrome on HPA axis response and peripheral glucocorticoid receptor expression. Psychoneuroendocrinology (2016) 69:67–76. 10.1016/j.psyneuen.2016.03.016 27038676 PMC4977028

[68] de Kloet CS Vermetten E Bikker A Meulman E Geuze E Kavelaars A Leukocyte glucocorticoid receptor expression and immunoregulation in veterans with and without post-traumatic stress disorder. Mol Psychiatry (2007) 12:443–53. 10.1038/sj.mp.4001934 17245326

[69] Yehuda R Golier JA Yang R-K Tischler L Enhanced sensitivity to glucocorticoids in peripheral mononuclear leukocytes in posttraumatic stress disorder. Biol Psychiatry (2004) 55:1110–6. 10.1016/j.biopsych.2004.02.010 15158431

[70] Yehuda R Flory JD Bierer LM Henn-Haase C Lehrner A Desarnaud F Lower methylation of glucocorticoid receptor gene promoter 1F in peripheral blood of veterans with posttraumatic stress disorder. Biol Psychiatry (2015) 77:356–64. 10.1016/j.biopsych.2014.02.006 24661442

[71] Gola H Engler A Morath J Adenauer H Elbert T Kolassa I-T Reduced peripheral expression of the glucocorticoid receptor α isoform in individuals with posttraumatic stress disorder: a cumulative effect of trauma burden. PLoS One (2014) 9:e86333. 10.1371/journal.pone.0086333 24466032 PMC3897679

[72] Hepgul N Cattaneo A Zunszain PA Pariante CM Depression pathogenesis and treatment: what can we learn from blood mRNA expression? BMC Med (2013) 11:28. 10.1186/1741-7015-11-28 23384232 PMC3606439

[73] Liew C-C Ma J Tang H-C Zheng R Dempsey AA The peripheral blood transcriptome dynamically reflects system wide biology: a potential diagnostic tool. J Lab Clin Med (2006) 147:126–32. 10.1016/j.lab.2005.10.005 16503242

[74] Lennon EM Maharshak N Elloumi H Borst L Plevy SE Moeser AJ Early life stress triggers persistent colonic barrier dysfunction and exacerbates colitis in adult IL-10-/- mice. Inflammation Bowel Dis (2013) 19:712–9. 10.1097/MIB.0b013e3182802a4e PMC4114389 23446335

[75] Santos J Benjamin M Yang PC Prior T Perdue MH Chronic stress impairs rat growth and jejunal epithelial barrier function: role of mast cells. Am J Physiol Gastrointest. Liver Physiol (2000) 278:G847–854. 10.1152/ajpgi.2000.278.6.G847 10859213

[76] Castagliuolo I Lamont JT Qiu B Fleming SM Bhaskar KR Nikulasson ST Acute stress causes mucin release from rat colon: role of corticotropin releasing factor and mast cells. Am J Physiol (1996) 271:G884–892. 10.1152/ajpgi.1996.271.5.G884 8944704

[77] Hoffman JM Baritaki S Ruiz JJ Sideri A Pothoulakis C Corticotropin-Releasing Hormone Receptor 2 Signaling Promotes Mucosal Repair Responses after Colitis. Am J Pathol (2016) 186:134–44. 10.1016/j.ajpath.2015.09.013 PMC4714033 26597886

[78] Moss AC Anton P Savidge T Newman P Cheifetz AS Gay J Urocortin II mediates pro-inflammatory effects in human colonocytes *via* corticotropin-releasing hormone receptor 2alpha. Gut (2007) 56:1210–7. 10.1136/gut.2006.110668 PMC1954994 17412781

[79] Bertiaux-Vandaële N Youmba SB Belmonte L Lecleire S Antonietti M Gourcerol G The expression and the cellular distribution of the tight junction proteins are altered in irritable bowel syndrome patients with differences according to the disease subtype. Am J Gastroenterol (2011) 106:2165–73. 10.1038/ajg.2011.257 22008894

[80] Dunlop SP Hebden J Campbell E Naesdal J Olbe L Perkins AC Abnormal intestinal permeability in subgroups of diarrhea-predominant irritable bowel syndromes. Am J Gastroenterol (2006) 101:1288–94. 10.1111/j.1572-0241.2006.00672.x 16771951

[81] Zhou Q Zhang B Verne GN Intestinal membrane permeability and hypersensitivity in the irritable bowel syndrome. Pain (2009) 146:41–6. 10.1016/j.pain.2009.06.017 PMC2763174 19595511

[82] Marshall JK Thabane M Garg AX Clark W Meddings J Collins SM Intestinal permeability in patients with irritable bowel syndrome after a waterborne outbreak of acute gastroenteritis in Walkerton, Ontario. Aliment. Pharmacol Ther (2004) 20:1317–22. 10.1111/j.1365-2036.2004.02284.x 15606393

[83] O’Mahony L McCarthy J Kelly P Hurley G Luo F Chen K Lactobacillus and bifidobacterium in irritable bowel syndrome: symptom responses and relationship to cytokine profiles. Gastroenterology (2005) 128:541–51. 10.1053/j.gastro.2004.11.050 15765388

[84] Dinan TG Quigley EMM Ahmed SMM Scully P O’Brien S O’Mahony L Hypothalamic-Pituitary-Gut Axis Dysregulation in Irritable Bowel Syndrome: Plasma Cytokines as a Potential Biomarker? Gastroenterology (2006) 130:304–11. 10.1053/j.gastro.2005.11.033 16472586

[85] Clarke G Quigley EMM Cryan JF Dinan TG Irritable bowel syndrome: towards biomarker identification. Trends Mol Med (2009) 15:478–89. 10.1016/j.molmed.2009.08.001 19811951

[86] Gecse K Róka R Ferrier L Leveque M Eutamene H Cartier C Increased faecal serine protease activity in diarrhoeic IBS patients: a colonic lumenal factor impairing colonic permeability and sensitivity. Gut (2008) 57:591–9. 10.1136/gut.2007.140210 18194983

[87] Barbara G Wang B Stanghellini V de Giorgio R Cremon C Di Nardo G Mast cell-dependent excitation of visceral-nociceptive sensory neurons in irritable bowel syndrome. Gastroenterology (2007) 132:26–37. 10.1053/j.gastro.2006.11.039 17241857

[88] Piche T Barbara G Aubert P Bruley des Varannes S Dainese R Nano JL Impaired intestinal barrier integrity in the colon of patients with irritable bowel syndrome: involvement of soluble mediators. Gut (2009) 58:196–201. 10.1136/gut.2007.140806 18824556

[89] Bird A DNA methylation patterns and epigenetic memory. Genes Dev (2002) 16:6–21. 10.1101/gad.947102 11782440

[90] Kiefer JC Epigenetics in development. Dev Dyn. (2007) 236:1144–56. 10.1002/dvdy.21094 17304537

[91] Géranton SM Fratto V Tochiki KK Hunt SP Descending serotonergic controls regulate inflammation-induced mechanical sensitivity and methyl-CpG-binding protein 2 phosphorylation in the rat superficial dorsal horn. Mol Pain (2008) 4:35. 10.1186/1744-8069-4-35 18793388 PMC2553762

[92] Géranton SM Morenilla-Palao C Hunt SP A role for transcriptional repressor methyl-CpG-binding protein 2 and plasticity-related gene serum- and glucocorticoid-inducible kinase 1 in the induction of inflammatory pain states. J Neurosci (2007) 27:6163–73. 10.1523/JNEUROSCI.1306-07.2007 PMC6672147 17553988

[93] McGowan PO Sasaki A D’Alessio AC Dymov S Labonté B Szyf M Epigenetic regulation of the glucocorticoid receptor in human brain associates with childhood abuse. Nat Neurosci (2009) 12:342–8. 10.1038/nn.2270 PMC2944040 19234457

[94] Deaton AM Bird A CpG islands and the regulation of transcription. Genes Dev (2011) 25:1010–22. 10.1101/gad.2037511 PMC3093116 21576262

[95] Teodoridis JM Strathdee G Brown R Epigenetic silencing mediated by CpG island methylation: potential as a therapeutic target and as a biomarker. Drug Resist Update (2004) 7:267–78. 10.1016/j.drup.2004.06.005 15533764

[96] Patnaik S Anupriya null. Drugs Targeting Epigenetic Modifications and Plausible Therapeutic Strategies Against Colorectal Cancer. Front Pharmacol (2019) 10:588. 10.3389/fphar.2019.00588 31244652 PMC6563763

[97] Yang X Han H De Carvalho DD Lay FD Jones PA Liang G Gene body methylation can alter gene expression and is a therapeutic target in cancer. Cancer Cell (2014) 26:577–90. 10.1016/j.ccr.2014.07.028 PMC4224113 25263941

[98] Esteller M Epigenetics in cancer. N. Engl J Med (2008) 358:1148–59. 10.1056/NEJMra072067 18337604

[99] Denk F McMahon SB Chronic pain: emerging evidence for the involvement of epigenetics. Neuron (2012) 73:435–44. 10.1016/j.neuron.2012.01.012 PMC3996727 22325197

[100] Radley JJ Kabbaj M Jacobson L Heydendael W Yehuda R Herman JP Stress risk factors and stress-related pathology: neuroplasticity, epigenetics and endophenotypes. Stress (2011) 14:481–97. 10.3109/10253890.2011.604751 PMC3641164 21848436

[101] Vaiserman AM Epigenetic programming by early-life stress: Evidence from human populations. Dev Dyn. (2015) 244:254–65. 10.1002/dvdy.24211 25298004

[102] Hullar MAJ Fu BC Diet, the gut microbiome, and epigenetics. Cancer J (2014) 20:170–5. 10.1097/PPO.0000000000000053 PMC4267719 24855003

[103] Weaver ICG Cervoni N Champagne FA D’Alessio AC Sharma S Seckl JR Epigenetic programming by maternal behavior. Nat Neurosci (2004) 7:847–54. 10.1038/nn1276 15220929

[104] Labonte B Yerko V Gross J Mechawar N Meaney MJ Szyf M Differential glucocorticoid receptor exon 1(B), 1(C), and 1(H) expression and methylation in suicide completers with a history of childhood abuse. Biol Psychiatry (2012) 72:41–8. 10.1016/j.biopsych.2012.01.034 22444201

[105] Watkeys OJ Kremerskothen K Quidé Y Fullerton JM Green MJ Glucocorticoid receptor gene (NR3C1) DNA methylation in association with trauma, psychopathology, transcript expression, or genotypic variation: A systematic review. Neurosci Biobehav Rev (2018) 95:85–122. 10.1016/j.neubiorev.2018.08.017 30176278

[106] Greenwood-Van Meerveld B Johnson AC Stress-Induced Chronic Visceral Pain of Gastrointestinal Origin. Front Syst Neurosci (2017) 11:86. 10.3389/fnsys.2017.00086 29213232 PMC5702626

[107] Perroud N Paoloni-Giacobino A Prada P Olié E Salzmann A Nicastro R Increased methylation of glucocorticoid receptor gene (NR3C1) in adults with a history of childhood maltreatment: a link with the severity and type of trauma. Transl Psychiatry (2011) 1:e59. 10.1038/tp.2011.60 22832351 PMC3309499

[108] Tran L Chaloner A Sawalha AH Greenwood Van-Meerveld B Importance of epigenetic mechanisms in visceral pain induced by chronic water avoidance stress. Psychoneuroendocrinology (2013) 38:898–906. 10.1016/j.psyneuen.2012.09.016 23084728

[109] Hong S Zheng G Wiley JW Epigenetic regulation of genes that modulate chronic stress-induced visceral pain in the peripheral nervous system. Gastroenterology (2015) 148:148–157.e7. 10.1053/j.gastro.2014.09.032 25263804 PMC4274248

[110] Mahurkar S Polytarchou C Iliopoulos D Pothoulakis C Mayer EA Chang L Genome-wide DNA methylation profiling of peripheral blood mononuclear cells in irritable bowel syndrome. Neurogastroenterol. Motil (2016) 28:410–22. 10.1111/nmo.12741 PMC4760882 26670691

[111] Grondona JM Hoyo-Becerra C Visser R Fernández-Llebrez P López-Ávalos MD The subcommissural organ and the development of the posterior commissure. Int Rev Cell Mol Biol (2012) 296:63–137. 10.1016/B978-0-12-394307-1.00002-3 22559938

[112] Richter HG Tomé MM Yulis CR Vío KJ Jiménez AJ Pérez-Fígares JM Transcription of SCO-spondin in the subcommissural organ: evidence for down-regulation mediated by serotonin. Brain Res Mol Brain Res (2004) 129:151–62. 10.1016/j.molbrainres.2004.07.003 15469891

[113] Hunter A Spechler PA Cwanger A Song Y Zhang Z Ying G DNA Methylation Is Associated with Altered Gene Expression in AMD. Invest Ophthalmol Vis Sci (2012) 53:2089–105. 10.1167/iovs.11-8449 PMC4108280 22410570

[114] Leygo C Williams M Jin HC Chan MWY Chu WK Grusch M DNA Methylation as a Noninvasive Epigenetic Biomarker for the Detection of Cancer. Dis Markers (2017) 2017:3726595. 10.1155/2017/3726595 29038612 PMC5605861

[115] Zhu S Min L Guo Q Li H Yu Y Zong Y Transcriptome and methylome profiling in a rat model of irritable bowel syndrome induced by stress. Int J Mol Med (2018) 42:2641–9. 10.3892/ijmm.2018.3823 PMC6192760 30106160

[116] Mahurkar-Joshi S Videlock EJ Iliopoulos D Pothoulakis C Mayer EA Chang L Epigenetic Changes in Blood Cells and Colonic Mucosa are Associated with Irritable Bowel Syndrome (IBS). Gastroenterology (2018) 154:S–214. 10.1016/S0016-5085(18)31105-3

[117] Hake SB Xiao A Allis CD Linking the epigenetic “language” of covalent histone modifications to cancer. Br J Cancer (2004) 90:761–9. 10.1038/sj.bjc.6601575 PMC2410168 14970850

[118] Bannister AJ Kouzarides T Regulation of chromatin by histone modifications. Cell Res (2011) 21:381–95. 10.1038/cr.2011.22 PMC3193420 21321607

[119] Bai G Wei D Zou S Ren K Dubner R Inhibition of class II histone deacetylases in the spinal cord attenuates inflammatory hyperalgesia. Mol Pain (2010) 6:51. 10.1186/1744-8069-6-51 20822541 PMC2942827

[120] Imai S Ikegami D Yamashita A Shimizu T Narita M Niikura K Epigenetic transcriptional activation of monocyte chemotactic protein 3 contributes to long-lasting neuropathic pain. Brain (2013) 136:828–43. 10.1093/brain/aws330 23364351

[121] Imai S Ikegami D Yamashita A Shimizu T Narita M Niikura K Epigenetic transcriptional activation of monocyte chemotactic protein 3 contributes to long-lasting neuropathic pain. Brain (2013) 136:828–43. 10.1093/brain/aws330 23364351

[122] Moloney RD Stilling RM Dinan TG Cryan JF Early-life stress-induced visceral hypersensitivity and anxiety behavior is reversed by histone deacetylase inhibition. Neurogastroenterol. Motil (2015) 27:1831–6. 10.1111/nmo.12675 26403543

[123] Moloney RD Johnson AC O’Mahony SM Dinan TG Greenwood-Van Meerveld B Cryan JF Stress and the Microbiota-Gut-Brain Axis in Visceral Pain: Relevance to Irritable Bowel Syndrome. CNS Neurosci Ther (2016) 22:102–17. 10.1111/cns.12490 PMC6492884 26662472

[124] Hong S Zheng G Wiley JW Epigenetic regulation of genes that modulate chronic stress-induced visceral pain in the peripheral nervous system. Gastroenterology (2015) 148:148–157.e7. 10.1053/j.gastro.2014.09.032 25263804 PMC4274248

[125] Aguirre JE Winston JH Sarna SK Neonatal immune challenge followed by adult immune challenge induces epigenetic-susceptibility to aggravated visceral hypersensitivity. Neurogastroenterol. Motil (2017) 29:125. 10.1111/nmo.13081 PMC7048321 28439935

[126] Filipowicz W Bhattacharyya SN Sonenberg N Mechanisms of post-transcriptional regulation by microRNAs: are the answers in sight? Nat Rev Genet (2008) 9:102–14. 10.1038/nrg2290 18197166

[127] Kress M Hüttenhofer A Landry M Kuner R Favereaux A Greenberg D microRNAs in nociceptive circuits as predictors of future clinical applications. Front Mol Neurosci (2013) 6:33. 10.3389/fnmol.2013.00033 24151455 PMC3798051

[128] Videlock EJ Mahurkar-Joshi S Iliopoulos D Pothoulakis C Meyer EA Chang L Dysregulation of the long-noncoding RNA, GHRLOS, in irritable bowel syndrome. Gastroenterology (2017) 152:S722. 10.1016/S0016-5085(17)32511-8

[129] Kapeller J Houghton LA Mönnikes H Walstab J Möller D Bönisch H First evidence for an association of a functional variant in the microRNA-510 target site of the serotonin receptor-type 3E gene with diarrhea predominant irritable bowel syndrome. Hum Mol Genet (2008) 17:2967–77. 10.1093/hmg/ddn195 18614545

[130] Fourie NH Peace RM Abey SK Sherwin LB Rahim-Williams B Smyser PA Elevated circulating miR-150 and miR-342-3p in patients with irritable bowel syndrome. Exp Mol Pathol (2014) 96:422–5. 10.1016/j.yexmp.2014.04.009 PMC4119883 24768587

[131] Gheinani AH Burkhard FC Monastyrskaya K Deciphering microRNA code in pain and inflammation: lessons from bladder pain syndrome. Cell Mol Life Sci (2013) 70:3773–89. 10.1007/s00018-013-1275-7 PMC11113193 23463234

[132] Pekow JR Kwon JH MicroRNAs in inflammatory bowel disease. Inflammation Bowel Dis (2012) 18:187–93. 10.1002/ibd.21691 PMC4169145 21425211

[133] Zhou Q Souba WW Croce CM Verne GN MicroRNA-29a regulates intestinal membrane permeability in patients with irritable bowel syndrome. Gut (2010) 59:775–84. 10.1136/gut.2009.181834 PMC2891786 19951903

[134] Randomised placebo-controlled trial of dietary glutamine supplements for postinfectious irritable bowel syndrome. Gut. Available at: https://gut.bmj.com/content/68/6/996 (Accessed December 20, 2019). 10.1136/gutjnl-2017-315136 PMC9549483 30108163

[135] Zhou Q Costinean S Croce CM Brasier AR Merwat S Larson SA MicroRNA 29 targets nuclear factor-κB-repressing factor and Claudin 1 to increase intestinal permeability. Gastroenterology (2015) 148:158–169.e8. 10.1053/j.gastro.2014.09.037 25277410 PMC4303568

[136] Martinez C Rodino-Janeiro BK Lobo B Stanifer ML Klaus B Granzow M miR-16 and miR-125b are involved in barrier function dysregulation through the modulation of claudin-2 and cingulin expression in the jejunum in IBS with diarrhoea. Gut (2017) 66:1537–8. 10.1136/gutjnl-2016-311477 PMC5561373 28082316

[137] Hou Q Huang Y Zhu S Li P Chen X Hou Z MiR-144 Increases Intestinal Permeability in IBS-D Rats by Targeting OCLN and ZO1. Cell Physiol Biochem (2017) 44:2256–68. 10.1159/000486059 29258088

[138] Hou Q Huang Y Zhang C Zhu S Li P Chen X MicroRNA-200a Targets Cannabinoid Receptor 1 and Serotonin Transporter to Increase Visceral Hyperalgesia in Diarrhea-predominant Irritable Bowel Syndrome Rats. J Neurogastroenterol. Motil (2018) 24:656–68. 10.5056/jnm18037 PMC6175558 30347941

[139] Liao X-J Mao W-M Wang Q Yang G-G Wu W-J Shao S-X MicroRNA-24 inhibits serotonin reuptake transporter expression and aggravates irritable bowel syndrome. Biochem Biophys Res Commun (2016) 469:288–93. 10.1016/j.bbrc.2015.11.102 26631964

[140] Wohlfarth C Schmitteckert S Härtle JD Houghton LA Dweep H Fortea M miR-16 and miR-103 impact 5-HT 4 receptor signalling and correlate with symptom profile in irritable bowel syndrome. Sci Rep (2017) 7:1–14. 10.1038/s41598-017-13982-0 29089619 PMC5665867

[141] Merhautova J Demlova R Slaby O MicroRNA-Based Therapy in Animal Models of Selected Gastrointestinal Cancers. Front Pharmacol (2016) 7:329. 10.3389/fphar.2016.00329 27729862 PMC5037200

[142] Chen L-L Linking Long Noncoding RNA Localization and Function. Trends Biochem Sci (2016) 41:761–72. 10.1016/j.tibs.2016.07.003 27499234

[143] Yarani R Mirza AH Kaur S Pociot F The emerging role of lncRNAs in inflammatory bowel disease. Exp Mol Med (2018) 50. 10.1038/s12276-018-0188-9 PMC6283835 30523244

[144] Miro-Blanch J Yanes O Epigenetic Regulation at the Interplay Between Gut Microbiota and Host Metabolism. Front Genet (2019) 10:638. 10.3389/fgene.2019.00638 31338107 PMC6628876

[145] Bouchard L Rabasa-Lhoret R Faraj M Lavoie M-E Mill J Pérusse L Differential epigenomic and transcriptomic responses in subcutaneous adipose tissue between low and high responders to caloric restriction. Am J Clin Nutr (2010) 91:309–20. 10.3945/ajcn.2009.28085 19939982

[146] Rea K O’Mahony SM Dinan TG Cryan JF The Role of the Gastrointestinal Microbiota in Visceral Pain. In: Greenwood-Van Meerveld B, editor. Gastrointestinal Pharmacology Handbook of Experimental Pharmacology. Cham: Springer International Publishing (2017). p. 269–87. 10.1007/164_2016_115 28035535

[147] Stilling RM Dinan TG Cryan JF Microbial genes, brain & behaviour – epigenetic regulation of the gut–brain axis. Genes Brain Behav (2014) 13:69–86. 10.1111/gbb.12109 24286462

[148] Russell WR Hoyles L Flint HJ Dumas M-E Colonic bacterial metabolites and human health. Curr Opin Microbiol. (2013) 16:246–54. 10.1016/j.mib.2013.07.002 23880135

[149] Martin CR Osadchiy V Kalani A Mayer EA The Brain-Gut-Microbiome Axis. Cell Mol Gastroenterol. Hepatol. (2018) 6:133–48. 10.1016/j.jcmgh.2018.04.003 PMC6047317 30023410

[150] Maslowski KM Mackay CR Diet, gut microbiota and immune responses. Nat Immunol (2011) 12:5–9. 10.1038/ni0111-5 21169997

[151] Krautkramer KA Rey FE Denu JM Chemical signaling between gut microbiota and host chromatin: What is your gut really saying? J Biol Chem (2017) 292:8582–93. 10.1074/jbc.R116.761577 PMC5448088 28389558

[152] Vaiopoulou A Karamanolis G Psaltopoulou T Karatzias G Gazouli M Molecular basis of the irritable bowel syndrome. World J Gastroenterol (2014) 20:376–83. 10.3748/wjg.v20.i2.376 PMC3923013 24574707

[153] DeBusk RM Fogarty CP Ordovas JM Kornman KS Nutritional genomics in practice: where do we begin? J Am Diet Assoc (2005) 105:589–98. 10.1016/j.jada.2005.01.002 15800562

[154] de Roest RH Dobbs BR Chapman BA Batman B O’Brien LA Leeper JA The low FODMAP diet improves gastrointestinal symptoms in patients with irritable bowel syndrome: a prospective study. Int J Clin Pract (2013) 67:895–903. 10.1111/ijcp.12128 23701141

[155] Halmos EP Power VA Shepherd SJ Gibson PR Muir JG A diet low in FODMAPs reduces symptoms of irritable bowel syndrome. Gastroenterology (2014) 146:67–75.e5. 10.1053/j.gastro.2013.09.046 24076059

[156] Coutinho SV Plotsky PM Sablad M Miller JC Zhou H Bayati AI Neonatal maternal separation alters stress-induced responses to viscerosomatic nociceptive stimuli in rat. Am J Physiol Gastrointest. Liver Physiol (2002) 282:G307–316. 10.1152/ajpgi.00240.2001 11804852

[157] Kelly TK De Carvalho DD Jones PA Epigenetic Modifications as Therapeutic Targets. Nat Biotechnol (2010) 28:1069–78. 10.1038/nbt.1678 PMC3022972 20944599

[158] Welting O Van Den Wijngaard RM De Jonge WJ Holman R Boeckxstaens GE Assessment of visceral sensitivity using radio telemetry in a rat model of maternal separation. Neurogastroenterol. Motil (2005) 17:838–45. 10.1111/j.1365-2982.2005.00677.x 16336499

